# The Impact of Neutrophil Recruitment to the Skin on the Pathology Induced by *Leishmania* Infection

**DOI:** 10.3389/fimmu.2021.649348

**Published:** 2021-03-01

**Authors:** Katiuska Passelli, Oaklyne Billion, Fabienne Tacchini-Cottier

**Affiliations:** Department of Biochemistry, WHO Collaborative Centre for Research and Training in Immunology, University of Lausanne, Lausanne, Switzerland

**Keywords:** skin, keratinocytes, wound healing, neutrophils, *Leishmania*, cutaneous leishmaniasis, visceral leishmaniasis, leishmaniasis

## Abstract

*Leishmania* (*L*.) are obligate intracellular protozoan parasites that cause the leishmaniases, a spectrum of neglected infectious vector-borne diseases with a broad range of clinical manifestations ranging from local cutaneous, to visceral forms of the diseases. The parasites are deposited in the mammalian skin during the blood meal of an infected female phlebotomine sand fly. The skin is a complex organ acting as the first line of physical and immune defense against pathogens. Insults to skin integrity, such as that occurring during insect feeding, induces the local secretion of pro-inflammatory molecules generating the rapid recruitment of neutrophils. At the site of infection, skin keratinocytes play a first role in host defense contributing to the recruitment of inflammatory cells to the infected dermis, of which neutrophils are the first recruited cells. Although neutrophils efficiently kill various pathogens including *Leishmania*, several *Leishmania* species have developed mechanisms to survive in these cells. In addition, through their rapid release of cytokines, neutrophils modulate the skin microenvironment at the site of infection, a process shaping the subsequent development of the adaptive immune response. Neutrophils may also be recruited later on in unhealing forms of cutaneous leishmaniasis and to the spleen and liver in visceral forms of the disease. Here, we will review the mechanisms involved in neutrophil recruitment to the skin following *Leishmania* infection focusing on the role of keratinocytes in this process. We will also discuss the distinct involvement of neutrophils in the outcome of leishmaniasis.

## The Leishmaniases

The Leishmaniases are a group of neglected vector-borne diseases caused by protozoan parasites belonging to the *Leishmania (L.)* genus. Parasites are deposited into the mammalian host skin during the blood meal of infected female phlebotomine sand flies. At least twenty different *Leishmania* species can infect humans causing three main clinical manifestations including cutaneous, mucocutaneous and visceral forms (1, 2). The outcome of the disease depends on the infecting species along with host factors. Cutaneous leishmaniasis (CL) is the most common form characterized by the appearance of a skin ulcer at the sand fly bite site, usually on exposed body parts. Although skin lesions are most of the time self-healing and localized, they can leave seriously disfiguring and disabling life-long scars (3). The frequency of self-healing lesions depends, amongst other factors, on the *Leishmania* species. For instance, more than 75% of lesions caused by *L. mexicana* and the 60–70% caused by *L. major* heal within 3 months. In contrast, lesions caused by other New World *Leishmania* spp. such as *L. panamensis* and *L. braziliensis* take longer to heal, with an average frequency of only 35 and 10% that, respectively, self-heal after 3 months (4). Disseminated forms of CL have also been observed, where CL can manifest as multiple non-ulcerative nodules disseminating to the entire body (5). Mucocutaneous leishmaniasis (MCL) is a major CL complication that can manifest days to years following the cutaneous lesion. There are between 0.4 and 20% of CL cases in Brazil and Bolivia, respectively (6). The most endemic area for MCL is Latin America, where it results from infection with *L. braziliensis, L. panamensis, L. guyanensis*, and *L. amazonensis* (1, 7). MCL develops when the parasites migrate from the localized skin lesion to mucosal tissues of the nose, mouth and throat cavities, through lymphatics and blood vessels. This can lead to massive destruction of the oral or nasal mucosa and potentially become life-threatening (8). Visceral leishmaniasis (VL) is the deadliest form of the disease resulting from parasite dissemination from the skin to visceral organs, such as the spleen and the liver. This leads to organ dysfunction, fever, weight loss, and is usually fatal if left untreated (9). Between 700,000 and 1 million new leishmaniasis cases are reported each year across 100 countries (2, 10). The disease predominantly affects poor populations living in precarious hygiene and housing conditions (2). Several efforts have been made in order to develop a vaccine, but no effective and safe vaccine to prevent human leishmaniasis is currently available (11). Several drugs are available to treat leishmaniasis but many of them present toxicity with severe side effects and low efficacy, correlating with frequent treatment interruptions promoting the development of drug resistance (12). Currently, resistance has been reported for most of the drugs in use. It is, therefore, crucial to gain a better understanding of the local immune response to *Leishmania* infection with the long-term goal to develop anti-leishmanial drugs, which are efficient, less toxic, easy to administrate and affordable for emerging countries (13).

## The Skin and Parasite Entry

The skin acts as the first line of physical and immune defense against *Leishmania* parasites. The two main layers composing the skin are the epidermis and the dermis. The outer surface is the epidermis, characterized by overlapping layers of keratinocytes infiltrated by melanocytes. The dermis is composed of intermingled fibroblasts and extracellular matrix and is drained by blood and lymphatic vessels. The skin also includes a vast population of immune cells including Langerhans cells (skin resident dendritic cells), T lymphocytes and recruited inflammatory cells such as natural killers, macrophages, mast cells, dendritic and neutrophils, all of which participate to the immune defense against *Leishmania* infection (14).

Keratinocytes sense pathogens and initiate the inflammatory immune response. To do this, these epidermal cells possess several innate immune receptors able to sense invading microbes. These receptors include mostly Toll-like receptors (TLR), NOD-like receptors (NLR), RIG-I-like receptors (RLR), and C-type lectin receptors (CLR). Notably, keratinocytes express TLR1, TLR2, TLR4, TLR5, TLR6, and TLR10 on their cell surface, and TLR3, and TLR9 on the endosomal surface (15–22). The activation of the TLR-signaling pathways in keratinocytes mediates the secretion of pro-inflammatory cytokines and chemokines, which are mainly involved in the activation of the Th1 immune response and the recruitment of myeloid cells, including neutrophils (23). In response to pathogens, the activation of these receptors in keratinocytes enables the secretion of innate immune mediators participating in the skin immune response. Notably, in humans, keratinocytes generate antimicrobial peptides, including β-defensins (24, 25) and cathelicidins (26, 27). In addition to the direct killing of bacteria, fungi and viruses, these antimicrobial peptides can activate leukocytes. Furthermore, keratinocytes are a source of chemotactic mediators and cytokines, that enable the recruitment and activation of immune cells in the skin. For example, epidermal cells contribute to the recruitment of neutrophils in response to CXCL1 and CXCL8 release (28, 29). Keratinocytes produce cytokines such as tumor necrosis factor (TNF)-α, IL-1α, IL-1β, IL-6, IL-18, and IL-10 (29). Keratinocytes can also recruit effector T cells, following the secretion of CXCL9, CXCL10, CXCL11, and CCL20 (29).

## *Leishmania* Deposition in the Skin

Phlebotomine sand flies probe the exposed skin several times to find vessels and create a blood pool from which they feed. During this process, *Leishmania* parasites are deposited and they get in contact with keratinocytes. Although *Leishmania* are not internalized by keratinocytes (30–33), the parasites have been observed to interact with these cells (33). Several studies demonstrated that, during *Leishmania* infection, keratinocytes secrete factors that modulate the immune response. Indeed, *L. major* phosphoglycans, some of the major surface glycans of the parasite, trigger TLR2 in non-hematopoietic cells including keratinocytes, promoting the release of chemokines essential for early neutrophil recruitment (33). Scorza et al. showed that, in response to *L. infantum* but not *L. major*, human keratinocytes upregulate the expression of pro-inflammatory cytokines such as IL-6, CXCL8, TNFα, and IL-1β. Conversely, IL-4 expression was increased in keratinocytes exposed to *L. major* (32). The same study further showed that keratinocytes exposed to *L. infantum* released factors promoting parasite control in monocytes (32). In the same line, Ehrchen et al. showed an increased expression of cytokines and chemokines in mouse keratinocytes isolated from *L. major*-resistant but not susceptible mice (34). In visceral leishmaniasis patients, the expression of IL-10 in keratinocytes correlated with increased pathogenesis (35). Finally, the apoptosis of keratinocytes in CL correlated with skin ulceration, a process which relies on the Fas/TRAIL apoptotic pathway (36–38).

Notably, the study of Ehrchen et al. suggested that IL-4 produced by keratinocytes promoted the development of a protective Th1 immune response in C57BL/6 mice infected with *L. major* (34). In order to assess whether the IL-4 secreted by keratinocytes acted in an autocrine manner, mice with specific deletion of the IL-4Rα in keratinocytes were generated on the C57BL/6 and BALB/c genetic background, that are, respectively, resistant, or susceptible to *L. major* infection. C57BL/6 mice deficient for IL-4Rα in keratinocytes were able to develop a Th1 immune response and to heal their lesions following *L. major* infection. These data indicated that, in C57BL/6 mice, IL-4 signaling in keratinocytes is not required for the development of a protective Th1 immune response (39). In a similar manner, infection of BALB/c mice deficient for IL-4Rα in keratinocytes developed non-healing lesions characterized by a Th2 immune response, which was similar to those developed in WT BALB/c mice (40). These results showed that autocrine stimulation of keratinocytes by IL-4 is not involved in disease evolution following *L. major* infection.

The contribution of keratinocytes in the activation of antigen-specific T cells has also been reported. In this regard, studies documented that keratinocytes express MHCII, but are lacking the expression of CD80 and CD86 co-stimulatory molecules, that are essential for the priming of naïve T cells (41). Indeed, despite the ability of keratinocytes to support T cell proliferation, they fail to activate naïve T cells and they induce T cell anergy (42, 43). The expression of the costimulatory molecule B7.2 was shown to be downregulated in keratinocytes from resistant but not susceptible mice infected with *L. major* (31). More recent data have shown that keratinocytes can process antigens and present them to antigen-specific CD4 and CD8 T cells, leading to cytokine production (44). However, the role of these interactions during *Leishmania* infection remains to be investigated.

In the skin, a heterogeneous population of resident immune cells, maintains the homeostasis and is critical for host defense. In addition to the epidermal layer described above, insults to epidermis and dermis integrity lead to the recruitment of circulating immune cells including dendritic cells, T cells, natural killer cells, monocytes and neutrophils. Amongst these, neutrophils are the first cells massively recruited to the damaged skin. We shall focus on the importance of neutrophils in the response to infection.

## Neutrophils and *Leishmania*

### Neutrophil General Functions

Neutrophils are the most abundant leukocytes in the human blood circulation. They can be rapidly recruited to sites of injury or infection and are major players in innate immune defense against various pathogens (45). At the site of infection, neutrophils phagocytose microorganisms and kill them using a variety of mechanisms. The cytoplasm of neutrophils is rich in pre-stored granules that contain microbicidal proteins that can be rapidly released in the phagosomes or into the local microenvironment in order to eliminate pathogens. Neutrophil activation also triggers a respiratory burst leading to the production of reactive oxygen species (ROS) that is toxic for microorganisms (46, 47). Moreover, neutrophils can extrude neutrophil extracellular traps (NETs), which are extracellular web-like fibers consisting of chromatin associated with antimicrobial granule proteins. NETs trap microorganisms, a process that reduces parasite spreading. They can also kill pathogens through their association with a high concentration of microbicidal components (48).

In addition to their primary killing functions, neutrophils are increasingly reported to play significant immunoregulatory roles. Indeed, they can secrete a vast repertoire of cytokines or chemokines that may impact on the recruitment and function of various cell types. Through interactions with other immune cells, neutrophils can also contribute to the orchestration of the adaptive immune response (49, 50).

Neutrophils are the first cells to be recruited when the skin barrier is injured. Their importance in limiting microbial dissemination is highlighted by the predisposition of patients with neutropenia, or defective neutrophils, to harbor severe bacterial, parasitic or invasive fungal infections (51, 52). In contrast to these protective functions, neutrophils can also induce important tissue damage and inflammation that need to be tightly controlled (53). Deregulated neutrophil function is a feature of a heterogeneous group of skin pathologies named neutrophilic dermatosis (ND). These are conditions characterized by a wide spectrum of cutaneous lesions due to accumulation of neutrophils in the skin. ND are mainly caused by genetic mutations leading to excessive activity or production of inflammatory meditators involved in neutrophil recruitment and activation (54, 55).

Neutrophils are also major players engaged upon tissue injury and they also actively contribute to wound healing. However, neutrophils can also exert a negative impact on wound healing in some contexts. They can for instance contribute to the development of non-healing diabetic wounds. It was demonstrated that diabetes primes neutrophils to produce NETs, which impair healthy tissue healing (56, 57). Neutrophils were also recently shown to contribute to the pathogenesis of leprosy, a process contributing to the formation of skin lesions and lesions of peripheral nerves observed in this disease (58).

## The Mechanisms Involved in Early Neutrophil Recruitment to the Skin Following *Leishmania* Infection

Neutrophils are rapidly recruited to the site of *Leishmania* inoculation during infection. The infiltration of these cells is regulated by intertwined mechanisms related to the initial skin tissue damage caused by sand flies probing for blood (59). Several studies using experimental mouse models have shown that neutrophils are recruited following natural infection with sand flies (60–62) and that several factors contribute to this process. These include salivary gland components and the promastigote secretory gel that is synthesized by *Leishmania* in the sand fly (63–65). Furthermore, recent data showed that sand fly gut bacteria induce IL-1β secretion, a cytokine that also contributes to the recruitment of neutrophils (62). Of note, neutrophil recruitment to the site of infection following natural infection is more sustained compared to intradermal inoculation of parasites by needle injection (60, 61).

Neutrophils were similarly observed to be rapidly recruited when *Leishmania* are needle inoculated in mice (33, 60, 66–75). Phosphate buffer saline (PBS) or parasite injection promoted similar neutrophil recruitment during the first hour of injection, however, already 2 h after *L. major* or *L. mexicana* infection, the neutrophil infiltration became parasite-dependent (60, 71, 74). In experimental models of CL, a relatively high dose of *Leishmania* has been commonly injected either subcutaneously (s.c) in the footpad (66–68), or in more recent studies intradermally (i.d.) in the ear (33, 60, 69–75). Since the sand fly deposits the parasite in the host dermis, i.d. inoculation into the skin is closer to the natural infection and injection of a low dose of parasites (10^3^ or 10^4^) would be more related to the parasite load transmitted in natural infection (76). Interestingly, differences in neutrophil recruitment were observed depending on the site of infection. Intradermal injection led to a higher infiltration of neutrophils compared to s.c injection, while monocytes were more rapidly recruited following s.c. infection, indicating a site-dependent recruitment of myeloid cells in the skin (75, 77). Although most of the sand flies transmit a low dose of *Leishmania* parasites, needle injection of at least 10^5^ *Leishmania* is required to promote rapid parasite-dependent recruitment of neutrophils (70, 74, 76), further revealing the importance of sand fly derived components in the recruitment of these cells. Recently, it has been reported that depending on the distance of parasite deposition to the bite site, the predominant cells to contain parasites can be either neutrophils or dermal macrophages (78).

The group of Laskay first proposed that *Leishmania* parasites are silently transmitted to macrophages following phagocytosis of apoptotic and infected neutrophils in a model called the “Trojan Horse.” This way of entry promotes the persistence of the parasites and their subsequent propagation in the host (79, 80). This model was recently validated *in vivo* and few dermal macrophages were visualized acquiring parasites through phagocytosis of apoptotic and parasitized neutrophils. In the same context, depletion of neutrophils before the infection reduced the number of infected dermal macrophages (78).

In mice, the recruitment of neutrophils to the site of infection following sand fly bite, or needle injection of a high dose of parasites, was shown to be bimodal, with a first rapid wave peaking during the first day post-infection (p.i), returning to basal levels by the second or third day p.i. (33, 61, 67, 69–71, 73, 74). A second wave of neutrophils occurring approximately 1 week post *L. major* infection was also observed, at a time that correlates with the appearance of the lesion (71). Following *L. panamensis* infection, the second peak of neutrophils was observed several weeks p.i and was shown to be significantly stronger than the first one (81). A second wave of neutrophils was also shown to infiltrate the skin tissue 7 weeks post *L. major* infection (82), 5 weeks post-*L. major* co-infection with lymphocytic choriomeningitis virus (LCMV) (83), and neutrophils were observed in the skin of unhealing chronic *L. mexicana* lesions (84). The timing of the second wave of neutrophils is thus well documented and its timing likely varies depending on the virulence of the parasites, the parasite dose injected as well as the *Leishmania* spp.

In line with these studies, amastigotes were shown to infect both human and mouse neutrophils (84–86). Furthermore, following *L. mexicana* infection, not only are lesional neutrophils heavily infected, but a subset of neutrophils was shown to be permissive for parasite replication, suggesting that neutrophils may also serve as a replicating niche and/or safe temporary host target in chronic infection (84). Of interest, *L. major* amastigotes were not observed to replicate in C57BL/6 neutrophils (75), a difference likely linked to the distinct formation of a parasitophorous vacuole and/or to differences in the composition of the parasite surface in the two *Leishmania* spp.

Several skin chemotactic factors are responsible for the recruitment of neutrophils following *Leishmania* infection. For instance, the chemokines CXCL1 (KC) and CXCL2 (MIP-2) in mice and CXCL8 (IL-8) in humans, granulocyte chemotactic protein 2 (GCP-2/CXCL6) in mice and the cleaved complement C3 were all shown to promote neutrophil recruitment (59, 87–89). Additionally, during the second wave of neutrophils, IL-17 also contributes to their recruitment (90).

## Effector Function of Neutrophils During Leishmaniasis

Although neutrophils possess an arsenal of mechanisms to kill infecting pathogens, some species of *Leishmania* can survive inside these cells and use them as a safe shelter while some others are killed by neutrophils (74, 84, 86, 91–96). The mechanisms involved in parasite escape in neutrophils will not be discussed as they have been recently reviewed (97), but we will discuss the impact of neutrophil presence on the *Leishmania*-induced pathology.

**Figure 1:**
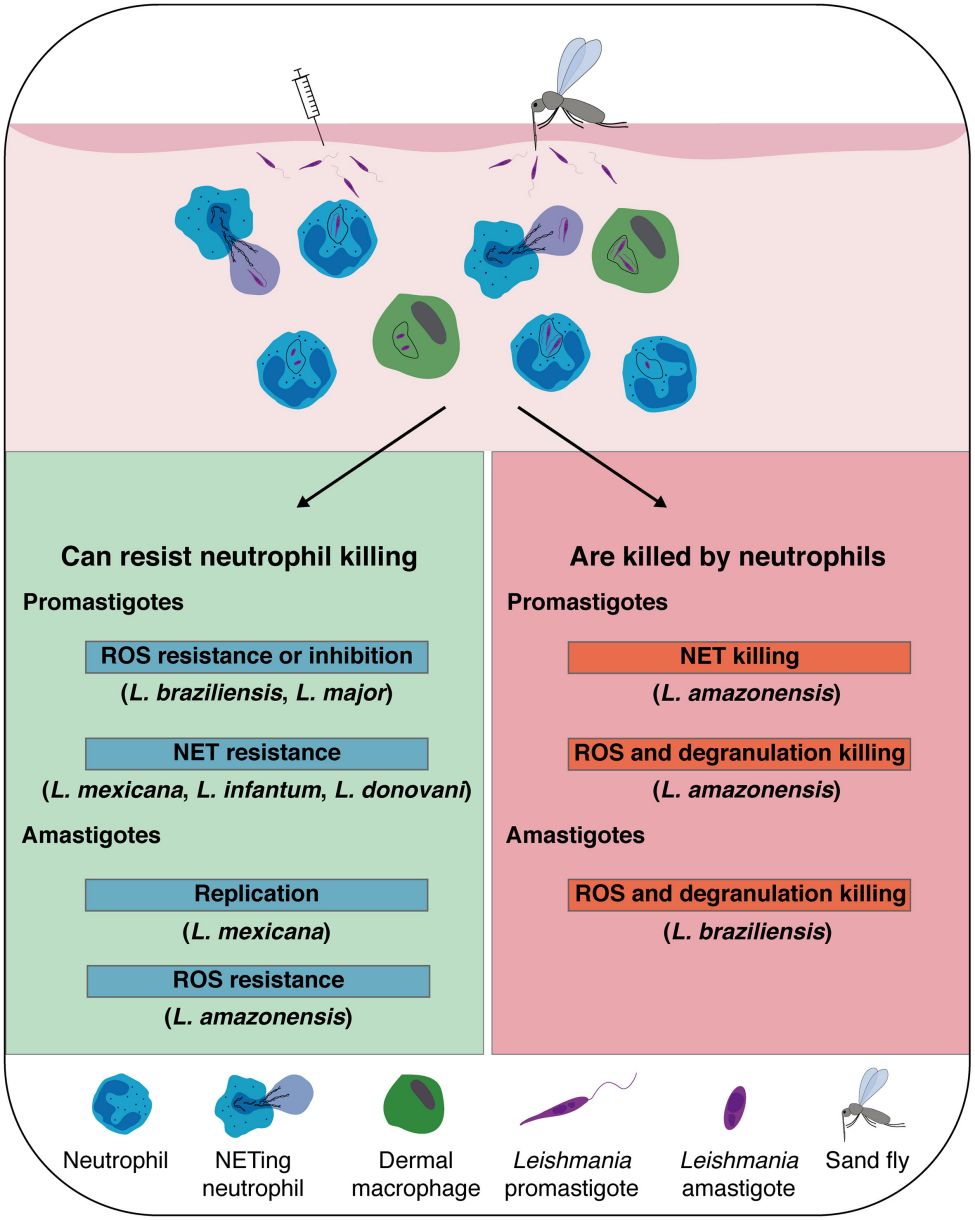
*Leishmania* spp. that are susceptible, or that escape neutrophil killing: *Leishmania* inoculation in the host dermis by natural sand fly bite, or experimental needle injection, promotes a rapid recruitment of neutrophils, which sequestrate the parasite by phagocytosis or release of neutrophil extracellular traps (NETs). In addition to neutrophils, dermal macrophages are also heavily infected early post-infection contributing to the establishment of the infection. Although neutrophil killing mechanisms efficiently eliminate some spp. of *Leishmania* promastigotes and amastigotes, some *Leishmania* developed mechanisms to resist or inhibit reactive oxygen species (ROS) formation and survive inside neutrophils and NETs. Notably, *L. mexicana* amastigotes were shown to replicate inside neutrophils.

The impact of neutrophils on the development of leishmaniasis has been observed to be either protective or detrimental depending on the infecting *Leishmania* species and the host genetics and immune system (Figure 1). Most of the current knowledge acquired related to the impact of neutrophils in experimental *Leishmania* infection has been obtained either following mAb-induced neutrophil depletion at the onset of infection or using genetically neutropenic mice (60, 67, 70, 72, 74, 82, 98–100). In order to transiently deplete neutrophils in mice, three monoclonal antibodies have been used, each of them with some limitations. Several studies have used the RB6-8RC5 monoclonal antibody that recognizes the Gr1 epitope which is common between cells expressing Ly6G and Ly6C (101). The RB6-8RC5 antibody efficiently depletes neutrophils and inflammatory monocytes (77). The NIMP-R14 mAb depletes neutrophils and also a subset of inflammatory monocytes (77, 102). Finally, the 1A8 monoclonal antibody targets specifically Ly6G that is expressed exclusively in neutrophils (103) but depletion is transient, and if used for more than a week, an increased release of neutrophils from the BM is observed. Differences in mAb isotypes also results in different depletion efficacy. Novel mAb administration strategies may circumvent this problem (104). One thus has to be critical in evaluating studies using only mAb depletion strategies. Complementary approaches abolishing or reducing the presence of neutrophils are needed to better understand the implications of these cells in the outcome of infection. Nevertheless, compiling results obtained with these different approaches completed by *in vitro* studies in human neutrophils allowed us to shed a new light on the importance of these cells in *Leishmania* pathogenicity, with either protective or pathogenic roles (Table 1).

**Table 1:** Protective and detrimental role of neutrophils during *Leishmania* infection: Neutrophils play protective (resolution of the disease), or detrimental (enhanced disease) roles in cutaneous and visceral leishmaniasis depending on the infecting *Leishmania* spp., the genetic background of the host, the mode of infection and the host immune response.

**Role of neutrophils in infection**	***Leishmania***		**Source of neutrophils**	**Route of infection**	**References**
	**species**	**strain**			
Protective	*L. major*	Bokkara	C57BL/6, BALB/c	s.c.^a^	(98)
		LV39	C57BL/6	s.c.	(67, 99)
		LV39	C57BL/6	i.d.^b^	(69, 75)
	*L. amazonensis*		BALB/c, C57BL/6	i.d.	(72, 105)
	*L. amazonensis*		BALB/c (Promastigote and amastigote parasites) C57BL/6, C3H/HePas, C3H/HeJ (promastigote parasites)	*in vitro*	(86, 106)
	*L. amazonensis*		Human	*in vitro*	(93, 115, 116)
	*L. braziliensis*		BALB/c	i.d.	(100)
	*L. braziliensis*		C57BL/6	*in vitro*	(73, 107)
	*L. donovani*		C57BL/6, BALB/c	i.v.^c^	(108, 109)
	*L. infantum*		C57BL/6, BALB/c	i.v. or i.d.	(70, 110–112)
Detrimental	*L. major*	LV39	BALB/c	s.c.	(67, 99)
		LV39	C57BL/6	i.d.	(33)
		IL81	Human	*in vitro*	(79)
		5ASKH	BALB/c	s.c.	(88)
		Friedlin	C57BL/6 (LCMV coinfection)	i.d.	(83)
		Friedlin	C57BL/6	i.d. (sand fly)	(60, 61)
		Friedlin	BALB/c	i.d	(90)
		Seidman	C57BL/6	i.d.	(82)
		Ryan	C57BL/6	i.d. (sand fly and needle)	(78)
	*L. mexicana*		C57BL/6, BALB/c	i.d.	(74, 114)
	*L. mexicana*		C57BL/6	i.d and *in vitro*	(84)
	*L. amazonensis*		C57BL/6 (amastigote parasites)	*in vitro*	(86)
	*L. panamensis*		Human	*ex vivo*	(118)
	*L. donovani*		BALB/c	i.d. (sand fly)	(62)

a *s.c.: subcutaneous in the footpad*.

b *i.d.: intradermal in the ear*.

c *i.v.: intravenous*.

*Light gray: CL; dark gray: VL. Parasites are needle injected. When parasites are deposited following natural infection, “sand fly” is indicated*.

## Protective Role for Neutrophils in Leishmaniasis

The first evidence suggesting a positive role of neutrophils during *Leishmania* infection was suggested in mice infected with the Bokkara strain of *L. major*. C57BL/6 mice depleted of neutrophils and monocytes (RB6-8RC5 antibody) at −3, 0 and 3 days post infection, and infected s.c. with 10^7^ *L. major*, showed increased lesion size and a higher parasite burden (98). Similarly, depletion of neutrophils using the NIMP-R14 antibody in C57BL/6 mice promoted the development of a more prominent lesion during the first weeks following *L. major* LV39 infection, but the lesion eventually healed (67). BALB/c mice infected with *L. major* Bokkara and depletion of neutrophils using the RB6-8RC5 antibody, showed increased lesion progression during the first 6 weeks post infection, without affecting the chronic progression of the disease (98). These latter results contrasted with other data (see below), and these differences might be explained by the distinct species or strains of parasite inoculated, different dose of parasite injected and the specificity of the antibodies used to deplete neutrophils.

Neutrophils were further reported to secrete CCL3, a chemokine that contributes to the recruitment of dendritic cells (DC) to the site of *L. major* infection in C57BL/6 mice, a process shown to contribute to the development of a protective immune response (69). In contrast, apoptotic neutrophils containing live *Leishmania* were shown to be uptaken by dermal dendritic cells, a process inhibiting the development of an adaptive immune response (71). These data further demonstrated a complex role for neutrophils in self-healing experimental CL. In addition, the sensing of *L. major* by neutrophil endosomal TLR7, was shown to be critical for the early regulation of parasite burden and subsequent disease control in *L. major* LV39 infected C57BL/6 mice (75).

The presence of neutrophils was also shown to be protective during the first week post-infection in BALB/c mice infected with *L. amazonensis* in a study using the RB6-8RC5 antibody to deplete neutrophils (72). *L. amazonensis* promastigotes were efficiently killed by neutrophils *in vitro*, whereas the amastigote form of the parasite resisted neutrophil killing (86), suggesting that neutrophils may have a distinct role early post-infection than during the chronic phase of the disease. In addition, following infection of mice with *L. amazonensis*, neutrophil programmed cell death was altered due to genetic defective ROS production, and pathology was shown to be associated with the presence of necrotic neutrophils, suggesting that appropriated neutrophil programmed cell death is essential for the control of *L. amazonensis*-induced dermal lesions (105). Similarly, the presence of neutrophils co-cultured *in vitro* with macrophages reduced *L. amazonensis* burden in macrophages (106). The depletion of neutrophils using the RB6-8RC5 antibody in BALB/c mice infected with *L. braziliensis*, also promoted the development of a more prominent lesion with increased parasite burden during the first 2 weeks post-infection (100). Moreover, Carlsen et al. showed that the internalization of *L. braziliensis* amastigotes by neutrophils induced a strong activation of these cells, leading to efficient killing of the parasite (107). Collectively, these data show that neutrophils are efficient in controlling the parasites following infection with these *Leishmania* spp.

Neutrophils were also shown to play a protective role following needle injection of *L. donovani* and *L. infantum*, two *Leishmania* spp. causing visceral leishmaniasis (VL). The infection of neutrophil-depleted C57BL/6 mice with *L. donovani* resulted in a hepatosplenomegaly and a higher parasite burden in the spleen and liver compared to neutrophil-sufficient mice. In this study, the RB6-8RC5 antibody was used for depletion purposes (108). In the same vein, neutrophils contributed to the development of a protective immune response during *L. donovani* infection of BALB/c mice. In this latter study, neutropenic mice had a splenomegaly with a higher parasite load in the spleen and bone marrow. This phenotype was associated with increased IL-4 and IL-10 secretion and reduced IFN-γ levels in the spleen whereby the NIMP-R14 antibody was used to deplete neutrophils (109). Additionally, using the RB6-8C5 depleting antibody, neutrophils were also shown to contribute to the early control of infection in BALB/c mice infected with *L. infantum* (70, 110). During *L. infantum* infection, TLR2 and TLR9 were shown to be critical for the recruitment of neutrophils to the inflammatory site, contributing to the control of infection (111, 112). Collectively, these studies show that, although the mAb used for neutrophil depletion was not always specific for neutrophils, neutrophils can play their well-known protective role in CL and VL.

## A Detrimental Role for Neutrophils During Leishmaniasis

The contribution of neutrophils in the development of a non-protective immunity was first reported using the classical *L. major* s.c. infection in BALB/c mice, that develop Th2 immune response correlating with the development of unhealing skin lesions. The transient depletion of neutrophils at the onset of infection in BALB/c mice, using the NIMP-R14 antibody, allowed better resolution of skin lesions and parasite burden, than mice injected with a control mAb, a process correlating with a decreased Th2 immune response (67). In addition, using the RB6-8RC5 antibody, Ribeiro-Gomes et al., also showed that neutrophil depletion reduced the parasite burden in *L. major-*infected BALB/c mice (99). Th17 cells secrete IL-17, a cytokine which recruits neutrophils. Following *L. major* infection, this IL-17-induced neutrophil recruitment was shown to promote CL pathogenicity in infected BALB/c mice (90).

Neutrophil recruitment to the skin also contributed to the severe pathology associated with infection with a low dose of *L. major* Seidman, a parasite that causes severe disease in C57BL/6 mice despite the development of a strong Th1 immune response (82, 113). Neutropenic *Genista* mice infected with *L. major* Seidman developed a healing lesion associated with a decreased parasite burden and an increased protective Th1 immune response, further emphasizing the role of neutrophils in influencing the adaptive immune response (82). Of note, the negative role of neutrophils was associated with the activation of the NLRP3 inflammasome and the subsequent secretion of IL-1β (82). Using a different approach, we recently showed that the recruitment of neutrophils induced by *L. major*-triggered TLR2 activation in non-hematopoietic cells, delayed the control of the disease in C57BL/6 mice infected with *L. major* LV39 (33).

Neutrophils contribute to the chronicity of *L. mexicana* disease in C57BL/6 mice and also play a negative role in the disease. This was demonstrated using mice either genetically neutropenic or depleted of neutrophils during the first days of infection after injection of the Ly6G mAb (74). Following i.d. infection with *L. mexicana*, neutropenic mice developed a small lesion that ultimately healed, in contrast to neutrophil sufficient mice that showed a persistent unhealing lesion. In addition, healing was shown to correlate with the development of a protective Th1 response and a decreased parasite burden (74). Furthermore, crosstalk between Th17 cells and neutrophils was shown to contribute to the enhanced susceptibility toward a chronic disease following *L. mexicana* infection in BALB/c mice (114). These studies further strengthen the role of neutrophils on the developing adaptive immune response. An important number of neutrophils has also been reported following co-infection of mice with *L. major* and LCMV and the development of chronic lesions highly infiltrated with neutrophils, correlated with the development of a more prominent lesion (83).

Finally, natural infection with *L. major* infected sand flies also showed that neutrophils contribute to the cutaneous pathology (60). Recently, the microbiota of sand flies was shown to be associated with neutrophil recruitment to the site of *L. donovani* infection. Treating the sand flies with antibiotics decreased neutrophil recruitment and impaired parasite visceralization, suggesting a pathological role for neutrophils in this model of infection (62). Of note these data contrast with the protective role for neutrophils in VL observed following needle injection of a high dose of parasites.

Collectively these studies suggest that, to firmly assign a role for neutrophils in *Leishmania* infection, several parameters need to be taken into account including the dose of infection, the site of parasite injection, the mode of parasite delivery, the genetic background and immune status of the host as well as the neutropenic model used.

## Neutrophils in Human Leishmaniasis

To understand the role of neutrophils upon *Leishmania* infection, mouse models have predominantly been used but an increasing number of studies has been performed using peripheral blood neutrophils *in vitro* or *ex vivo* and using transcriptomics studies of infected skin biopsies. Results from these studies also suggest a dual role for neutrophils in human leishmaniasis, that can also be beneficial or detrimental depending on the infecting *Leishmania* species.

## Protective Role of Human Neutrophils During *Leishmania* Infection

Human neutrophils infected *in vitro* with *L. amazonensis* were reported to be activated and to produce ROS. Moreover, infected human neutrophils were shown to produce leukotriene B4 (LTB4), which promotes neutrophil degranulation and the killing of the parasite (115). Neutrophil degranulation in response to *L. amazonensis* was further shown to promote the killing of the parasite in infected macrophages (116).

## Deleterious Role of Human Neutrophils During *Leishmania* Infection

The transcriptional profile of primary human macrophages infected *in vitro* with *L. (Vianna) panamensis*, causing tegumentary leishmaniasis, differed significantly in response to infection depending on whether infection was undertaken with parasites isolated from self-healing patients or those showing chronic disease. Interestingly, expression levels of neutrophil-recruiting chemokines were predominantly observed in the transcriptomics analysis of macrophages infected with parasites isolated from chronic lesions, suggesting that the presence of neutrophils likely contributes to the sustained inflammation observed in chronic dermal leishmaniasis (117). In addition, performing transcriptomics of infected skin biopsies of patients that cured or failed to heal after meglumine antimoniate treatment, further showed that downregulation of the neutrophil activation genes was linked to cure of the disease, further supporting a deleterious role for neutrophils in chronic forms of the disease (118).

Neutrophils have been detected in patients with active cutaneous lesions of American tegumentary leishmaniasis from *L. braziliensis* patients (119–121). Neutrophils isolated from disseminated leishmaniasis patients infected with *L. brazililensis* were shown to be less activated and may thus contribute to parasite survival and dissemination (122).

In patients with active VL the number of circulating blood neutrophils was reduced and a considerable proportion of these blood peripheral cells was immature (123). Furthermore, neutrophils from VL patients were shown to be activated but to display impaired effector functions, suggesting that during active disease, neutrophils may contribute to the immunosuppression observed in VL patients (123, 124). Saliva components from the sand fly vector were shown to induce IL-17-induced neutrophil recruitment, a process favoring *L*. *infantum* infection (125) revealing, as was previously shown in murine models, an important role for this cytokine in neutrophil recruitment.

## Conclusion and Perspectives

Neutrophils are rapidly recruited to the skin following *Leishmania* infection and they play a critical role in phagocytosing the parasites and in shaping the immune response. In addition, neutrophils are also present in established lesions and were shown to contribute to parasite persistence. The impact of neutrophils on the development of leishmaniasis can be either protective, or detrimental, depending on a range of intricate factors including the infecting *Leishmania* spp., the host genetic background and immune response, as well as the local microenvironment induced by the parasite inoculation into the skin. In the last decade, keratinocytes have been identified as important cells for the modulation of the immune response following *Leishmania* infection, including neutrophil recruitment, further highlighting the importance of skin resident cells in the response against infection.

Many questions on the mechanism of action of neutrophils in leishmaniasis remain open. These include the understanding of the interactions between neutrophils and mast cells at the site of infection, the mechanisms involved in neutrophil recruitment to the draining lymphoid organs following infection and the interactions taking place between neutrophils and other recruited and resident cells in these organs. Further studies will help to identify potential targets for the treatment of leishmaniasis, in which neutrophils are contributing to the severity of the disease.

## Author Contributions

KP and FT-C wrote the review. OB, KP, and FT-C contributed to the figure and the table. All authors gave input on the review.

## Conflict of Interest

The authors declare that the research was conducted in the absence of any commercial or financial relationships that could be construed as a potential conflict of interest.
